# Anti-Epidermal Growth Factor Receptor (EGFR) Antibodies Overcome Resistance of Ovarian Cancer Cells to Targeted Therapy and Natural Cytotoxicity

**DOI:** 10.3390/ijms130912000

**Published:** 2012-09-20

**Authors:** Nina Gottschalk, Rainer Kimmig, Stephan Lang, Mahavir Singh, Sven Brandau

**Affiliations:** 1 Department of Otorhinolaryngology, University of Duisburg-Essen, 45147 Essen, Germany; E-Mails: nina.gottschalk@uk-essen.de (N.G.); stephan.lang@uk-essen.de (S.L.); 2 Department of Gynecology and Obstetrics, University of Duisburg-Essen, 45157 Essen, Germany; E-Mail: rainer.kimmig@uk-essen.de; 3 Lionex Diagnostics and Therapeutics, 38126 Braunschweig, Germany; E-Mail: info@lionex.de

**Keywords:** ovarian cancer, NK cell, cetuximab, EGFR, PstS-1, antibody-dependent cellular cytotoxicity

## Abstract

The poor outcome of advanced ovarian cancer under conventional therapy stimulated the exploration of new strategies to improve therapeutic efficacy. In our preclinical *in vitro* study we investigated a combination of targeted therapy and immunotherapy. Combination treatment with the anti-EGFR-antibody Cetuximab, related tyrosine kinase inhibitors (TKI) and cytolytic NK cells was tested against different ovarian cancer cell lines and primary tumour cells cultured from patient ascites. We found that selected ovarian cancer cells were susceptible to cetuximab and anti-EGFR-TKI-treatment, while the majority of cell lines were resistant to single or combination treatment with both substances. In addition, most ovarian cancer cells displayed low susceptibility to natural cytotoxicity of unstimulated NK cells. Notably, NK cytotoxicity against resistant ovarian cancer cells could be effectively enhanced by addition of Cetuximab mediating antibody-dependent cellular cytotoxicity (ADCC). Neither natural cytotoxicity nor ADCC of NK cells were negatively affected by the presence of TKIs. ADCC could be further increased when NK cells were pre-stimulated with monocytes and the immunostimulatory mycobacterial protein PstS-1. Our data suggest that targeted antibody therapy could be beneficial even against resistant tumour cells by augmenting supplementary cytolytic NK functions. Future studies should evaluate the combination of targeted therapy and immunotherapeutic approaches in patients with advanced ovarian cancer being resistant to standard treatment.

## 1. Introduction

Ovarian cancer is still the most lethal gynecological malignancy. In the United States, about 22,280 new cases of ovarian cancer and 15,500 deaths were estimated in 2012 [1]. The majority of cases are diagnosed in advanced stages with 5-year survival rate of only 26.9% [1]. The first-line treatment with radical surgery debulking followed by platin-taxanes-based chemotherapy is highly effective. The poor long-term prognosis is due to recurrence and lack of effective second-line chemotherapeutic regimens. Thus, targeted therapies and novel immunotherapeutic approaches might improve clinical outcome.

The EGFR (epidermal growth factor receptor) has been suggested as a promising target since up to 70% of ovarian cancers are EGFR-positive and EGFR-overexpression is developed during cancer progression and correlated to poor prognosis [2,3]. The efficacy of EGFR-inhibition has been studied with monoclonal antibodies and low molecular weight tyrosine kinase inhibitors (TKI). The chimeric monoclonal anti-EGFR-antibody Cetuximab binds to the extracellular domain of the EGF-receptor, prevents EGFR-signaling and enhances receptor internalization. *In vitro* Cetuximab inhibited cell growth of ovarian cancer cell lines and acted synergistically with cytostatic agents [4]. Further, Cetuximab is able to potentiate apoptosis, to inhibit angiogenesis and impairs tumour cell invasion and metastasis [5]. However, in clinical trials Cetuximab has failed to show relevant clinical activity as monotherapy or in combination with chemotherapy in ovarian cancer so far [6–8]. Small molecules as tyrosine kinase inhibitors act intracellularly by competing with ATP binding and prevent further intracellular receptor signaling. In several phase I-II-studies of ovarian cancer the tyrosine kinase inhibitor Erlotinib (Tarceva^®^) did not effectively contribute to a therapeutic improvement neither as a single agent nor combined with chemotherapy or with the anti-VEGF-antibody Bevacizumab [9–12]. Single TKI-inhibition with Gefitinib (Iressa^®^) reached only limited responses [13,14]. Preclinical data revealed that Gefitinib could potentiate cytostatic antitumoural effects [15], which might be also of clinical benefit [16]. Vandetanib (ZD6474, Zactima™), which inhibits VEGFR2 and EGFR signaling, had no clinical activity in monotherapy in recurrent ovarian cancer [17]. These studies show that, so far, EGFR-targeting in ovarian cancer has not reached sufficient clinical benefit.

Beside inhibition of signaling pathways, anti-EGFR-targeted therapies might also exert immune modulating effects. In addition to their direct antitumoural activity monoclonal antibodies (mAbs) like Cetuximab are able to mediate antibody-dependent cellular cytotoxicity (ADCC). NK cells, monocytes and granulocytes lyse mAb-coated tumour cells after binding via Fcγ-receptors (FcγRs). This hypothesis is supported for example by the finding that clinical response to mAbs is correlated to certain polymorphisms of FcγRs [18]. NK cells identify altered cells by down regulated MHC class I-molecules (missing self-hypothesis) or recognize transformed cells by specific receptors (e.g., MICA/MICB, ULBPs). The activation of the corresponding cytotoxicity receptors NKp46, NKp44, NKp30 is dependent on further regulatory receptors (KIR’s, killer cell immunoglobulin like receptors, KLR’s, killer cell lectin receptors). Finally, target cells are lysed by the release of perforin/granzymes or induction of apoptosis via Fas/Fas ligand or TRAIL [19]. In contrast, TKI are not able to mediate ADCC due to their different mode of action. However, previous studies have shown that NK function can be impaired by the TKI Dasatinib and Nilotinib [20]. So far, there is no data available regarding NK function in the presence of the anti-EGFR-TKI’s Erlotinib, Gefitinib or Vandetanib.

In ovarian cancer clinical efficacy of anti-EGFR agents is limited by primary resistance or immune escape mechanisms. Thus, immunogenic substances like bacterial components might function as immune-enhancers. For example, apathogenic viable BCG (Bacillus Calmette-Guerin) mycobacteria were shown to be effective in local therapy of superficial bladder cancer [21], in which NK cells seemed to be the pivotal immune effector cells [22,23]. Further studies developed non-viable, molecularly defined immunotherapeutic proteins such as PstS-1, a 38kDa-preparation of the cell membrane of M. tuberculosis [24]. Its immunogenicity was apparent in strong B- and T cell response after immunization [25], making PstS-1 a valuable serodiagnostic tool. PstS-1 stimulated peripheral blood mononuclear cells (PBMC’s) and human dendritic cells resulting in antitumoural activity in bladder cancer and melanoma [26]. Activation of toll-like-receptor (TLR)-2 and TLR-4 involving ERK1/2 and MAPK-pathways with consecutive production of IL-6 and TNF-a might be the underlying mechanisms [27,28]. Previous studies in our own laboratory evaluated the immunostimulatory potential of PstS-1 in ovarian cancer. We could show that PstS-1 indirectly augmented NK cell function directed against ovarian cancer cells via activation of monocytes (Gottschalk *et al.*, submitted). However, the role of TKI’s in this immunotherapeutic model has not been elucidated yet.

The aim of this study was first to investigate the combined inhibition of EGFR by Cetuximab and the tyrosine kinase inhibitors Erlotinib, Gefitinib and Vandetanib in an ascites-derived primary cell culture and in a variety of ovarian cancer cell lines with different EGFR-expression. Secondly, we wanted to determine the potential modulation of NK cell-mediated cytolysis against ovarian cancer cells in the presence of pharmacologic EGFR-inhibition. Thirdly, we investigated the stimulatory potential of the immunogenic protein PstS-1 on NK-cytotoxicity in combination with monocytes and anti-EGFR agents.

## 2. Results and Discussion

### 2.1. Susceptibility of Ovarian Cancer Cells to the Anti-EGFR-Antibody Cetuximab

**Figure 1:**
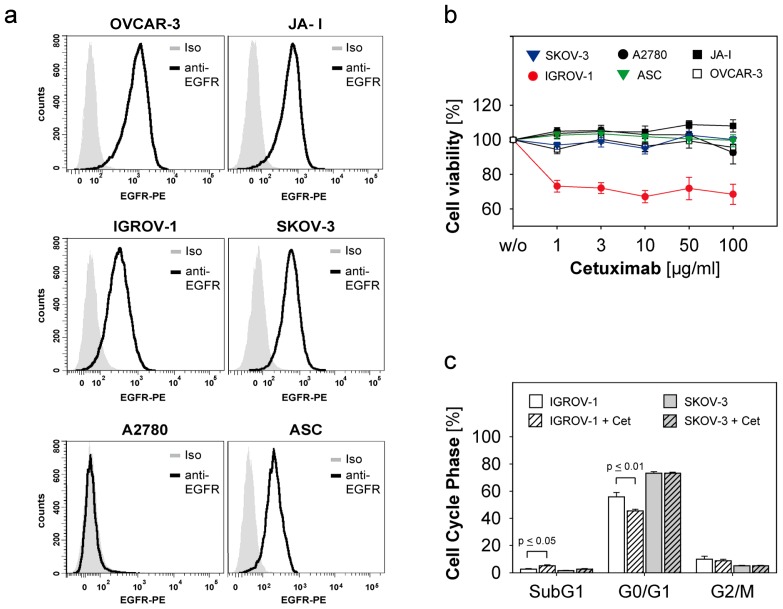
Epidermal growth factor receptor (EGFR)-expression of ovarian cancer cells and their susceptibility to Cetuximab. (**a**) The EGFR-expression of five different ovarian cancer cell lines and one primary ascites culture (ASC) was determined by flow cytometry after staining with PE-coupled anti-EGFR and isotype (Iso); (**b**) The ovarian cancer cells were seeded in 96-well-plates (10,000 cells/well) and treated with different concentrations of Cetuximab for 72 h at 37 °C. Cell viability was determined by the MTT-assay. Means and standard errors of three to seven independent experiments are shown; (**c**) Cell cycle was analyzed by flow cytometry after propidium iodide staining. Means and standard error of the percentage of cells in the respective cell cycle phases are shown for three independent experiments performed with IGROV-1 and SKOV-3. Statistical analysis was performed by unpaired *t*-test, statistical significance (*p* < 0.05) is indicated.

Five ovarian cancer cell lines IGROV-1, SKOV-3, A2780, OVCAR-3, JA-I and one primary ascites culture (ASC) were characterized regarding the expression of EGFR by flow cytometry as shown in Figure 1a. Thereby the strongest EGFR-expression was shown for OVCAR-3, followed by JA-I and SKOV-3. IGROV-1 and ASC showed moderate expression of EGFR, A2780 was EGFR-negative. The cell lines and ASC were coincubated with the anti-EGFR-antibody Cetuximab in concentrations of 1, 3, 10, 50 and 100 μg/mL for 72 h. We evaluated the antiproliferative effect of Cetuximab using the MTT-assay [29]. Changes in cell cycle were analyzed by flow cytometry after propidium iodide staining [30]. The only susceptible cell line was IGROV-1 showing reduced cell growth (Figure 1b). A significant decrease in the cell cycle phase G0/G1 (*p* ≤ 0.01) of IGROV-1 was observed, while the apoptotic fraction SubG1 was significantly increased (*p* ≤ 0.05) (Figure 1c). In contrast, SKOV-3 and the other ovarian cancer cells were not affected by Cetuximab-treatment (Figure 1b,c).

Our data confirm the previously described effects of Cetuximab on cell proliferation and cell cycle in susceptible cancer cells [31], but most ovarian cancer cells displayed functional resistance to cetuximab treatment despite high EGFR-expression. Several mechanisms like the presence of K-RAS-mutations or disturbed EGFR-internalization and –degradation with consecutive activation of other members of the HER-receptor-family might play a crucial role [32,33]. Our data are also in agreement with previous studies showing that high EGFR-expression does not predict susceptibility to Cetuximab [7]. In conclusion, in our studies Cetuximab seemed to be an effective anti-EGFR-agent only in a minority of ovarian cancer cell lines, which might correspond to the limited clinical efficacy of Cetuximab as monotherapy in ovarian cancer [8].

### 2.2. Response of Ovarian Cancer Cells to the Anti-EGFR-Tyrosine Kinase Inhibitors (TKI)

**Figure 2:**
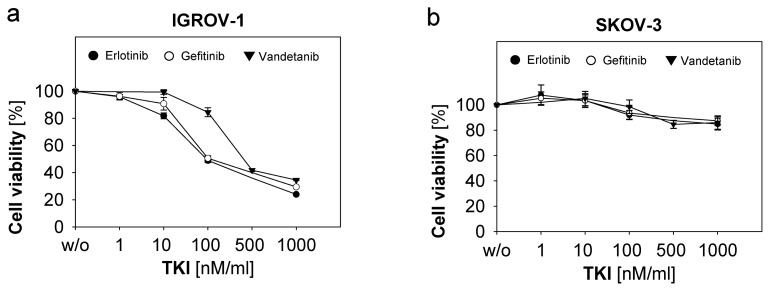

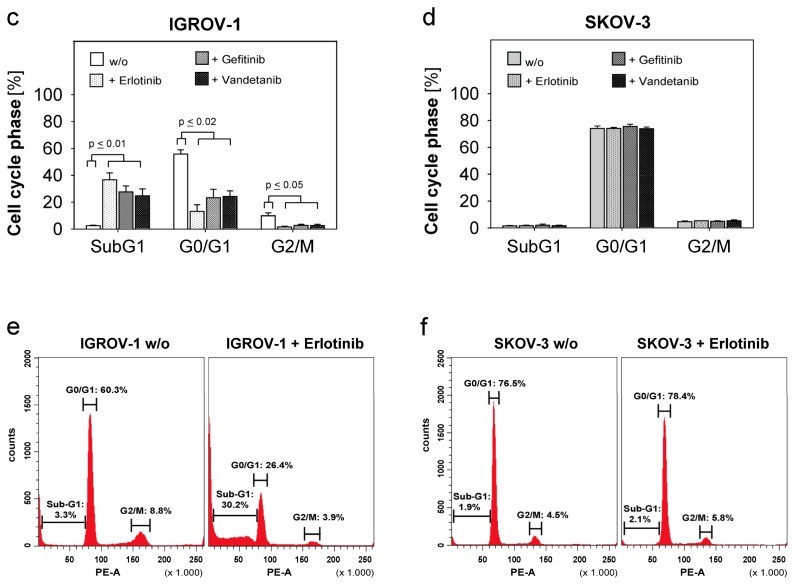
Susceptibility of ovarian cancer cells to anti-EGFR-tyrosine-kinase-inhibitors (TKI). Ovarian cancer cells were incubated with Erlotinib, Gefitinib and Vandetanib in the indicated concentrations for 72 h at 37 °C. Cell viability was determined by the MTT-assay. Means and standard errors of three to four independent experiments are shown for IGROV-1 (**a**) and SKOV-3 (**b**). Cell cycle analysis was performed. Means and standard errors of three independent experiments are shown in (**c**) and (**d**). Representative experiments of flow cytometric cell cycle analysis are depicted for IGROV-1 (**e**) and SKOV-3 (**f**) showing percentage of positive cells for each cell cycle phase. Statistical analysis was performed by unpaired *t*-test, statistical significance (*p* < 0.05) is indicated.

Next we investigated the response of ASC and the ovarian cancer cell lines described above to various anti-EGFR-tyrosine kinase inhibitors (TKI’s). We used Erlotinib and Gefitinib in the concentrations 1 μM, 100 nM, 10 nM and 1 nM and Vandetanib in a dose range of 1 μM down to 10 nM. Cell growth inhibition and effects on cell cycle phases were determined by MTT-assay and flow cytometry, respectively. IGROV-1 showed a strongly dose-dependent decrease of cell proliferation in the presence of all three TKI’s (Figure 2a). Major changes in cell cycle phases with increase of the apoptotic fraction SubG1 (*p* ≤ 0.01 for each TKI) and decrease of the G0/G1-phase (*p* ≤ 0.02 per TKI) and G2/M-phase (*p* ≤ 0.05 for each TKI) in response to all three TKIs (Figure 2c,e) were observed. SKOV-3 and the other cell lines including ASC remained unaffected by TKI-treatment as depicted for SKOV-3 regarding cell proliferation (Figure 2b) and cell cycle changes (Figure 2d,f). Our data show that resistance to anti-EGFR-TKI’s was paralleled by the resistance to cetuximab. In turn, susceptibility to Cetuximab predicted sensitivity to anti-EGFR-TKI-treatment.

Several *in vitro*-studies could demonstrate the antiproliferative and apoptosis-inducing effects of TKIs in human cancer cells [15]. Based on their intracellular mode of action TKIs should be able to overcome certain protein structure-dependent antibody-resistance mechanism. For example, mutations of the extracellular domain of EGFR, like type III mutation (EGFRvIII), frequently mediating Cetuximab-resistance might be abrogated by TKIs. Additionally, TKIs like Erlotinib and Gefitinib and much more dual tyrosine kinase inhibitors like Vandetanib show certain cross-reactivity for different tyrosine kinases. So, this complementary mode of action of TKIs might be favourable to overcome antibody-related resistance. However, in our studies Cetuximab-resistance was not abrogated by anti-EGFR-TKI, which correlated to findings in other studies [34]. The factors determining susceptibility to TKI-treatment seem to be more complex. There is evidence that in non-small-cell lung cancer mutations in the TK-domain are crucial and predictive for response to Gefitinib [35]. In ovarian cancer specific TK-domain-mutations are usually not so common [36], even if in a Japanese cohort a mutation-frequency of 23.5% was indicated [37]. Therefore, further studies are needed regarding the role of EGFR-TK-mutations and factors predicting susceptibility to anti-EGFR-TKI in ovarian cancer.

### 2.3. Combined Use of Anti-EGFR-Tyrosine Kinase Inhibitors (TKI) and Anti-EGFR-Antibody Cetuximab in Ovarian Cancer Cells

**Figure 3:**
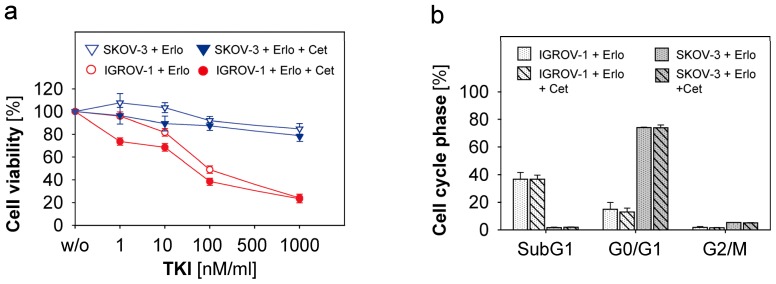
Susceptibility of ovarian cancer cells to combined treatment with Cetuximab and TKI. Ovarian cancer cells were treated with Erlotinib and Gefitinib (both 1 nM, 10 nM, 100 nM, 1 μM) and Vandetanib (10 nM, 100 nM, 500 nM and 1 μM). Where indicated in the legend Cetuximab was added in a concentration of 1 μg/mL. After incubation time of 72 h cell viability was determined by the MTT-assay. Means and standard error of three to four independent experiments are shown for IGROV-1 and SKOV-3 in (**a**). Cell cycle analysis of IGROV-1 and SKOV-3 after combined treatment is shown in (**b**). Means and standard errors of three independent experiments are indicated. Statistical analysis was performed by unpaired *t*-test.

The different mode of action of monoclonal antibodies and TKIs might suggest that the combined use of both substances might increase antitumoural activity. Therefore, we investigated whether the combined use of Erlotinib, Gefitinib and Vandetanib with Cetuximab might overcome resistance to the single agents. We assessed the cell viability and changes in cell cycle in IGROV-1, SKOV-3, A2780 and ASC. TKIs were concentrated as described above. Cetuximab was added in a concentration of 1 μg/mL. Cell viability and cell cycle analysis were performed after 72 h incubation time. As depicted in Figure 3a (red lines) in the anti-EGFR-susceptible cell line IGROV-1 cell proliferation was inhibited similar to the levels achieved by the mono-agents (compare Figures 1b and 2a). An additional or synergistic effect of combination therapy was not observed. Reduction of cell viability under high TKI-concentrations could not be further augmented by Cetuximab. The resistant ovarian cancer cells like SKOV-3 and ASC were not affected by combined treatment (Figure 3a for SKOV-3, blue lines). Accordingly, cell cycle studies of both susceptible IGROV-1 cells and resistant SKOV-3 cells revealed no difference between combination treatment *versus* treatment with mono-agents (Figure 3b). Our data suggest that a combination of TKI and Cetuximab is not superior to single agents alone. This is true for both resistant and susceptible cell lines.

This dual anti-EGFR-targeted therapy has not been described in ovarian cancer so far. However, there are in vitro studies regarding the growth control of hepatocellular cancer cells in which Cetuximab and Erlotinib exerted synergistic antitumoural effects [38]. In various squamous cell carcinoma cell lines growth inhibition by Cetuximab was enhanced by Erlotinib and Gefitinib, and Cetuximab-resistant cell lines remained susceptible for TKI treatment [39]. Clinical studies evaluating dual inhibition by Cetuximab and TKI are controversial. In lung cancer with acquired resistance to Erlotinib combined therapy failed to show activity [40], while in metastatic colorectal cancer recent results from a phase II-study seemed to be promising [41]. In ovarian cancer the role of combined targeted anti-EGFR-therapy remains unclear so far and needs further studies.

### 2.4. Combined Use of TKI, Cetuximab, Immune Effector Cells and Immunostimulatory Bacterial PstS-1

Even if monoclonal antibodies fail to exert direct antitumoural activity due to functional resistance of the target, they may contribute to tumour cell lysis by mediating antibody-dependent cellular cytotoxicity (ADCC). Unexpectedly, TKIs also may elicit immune-modulating function as it has been described for Dasatinib and Nilotinib [20]. Therefore, in the following experiments we focused on the capacity of NK cells to exert cytolytic activity against ovarian cancer cells, which are mainly resistant to Cetuximab and anti-EGFR-TKIs. To this end we examined the expression of CD107a on NK cells, which is expressed during degranulation and correlates with target cell lysis [42]. Further, we investigated whether addition of monocytes and immunostimulatory mycobacterial PstS-1 might further enhance NK-mediated cytotoxicity in the presence of Cetuximab and TKI.

**Figure 4:**
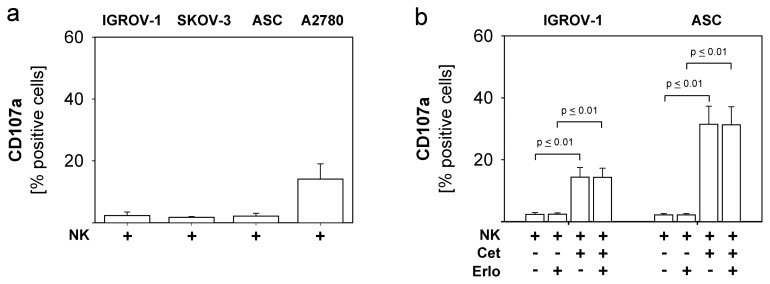

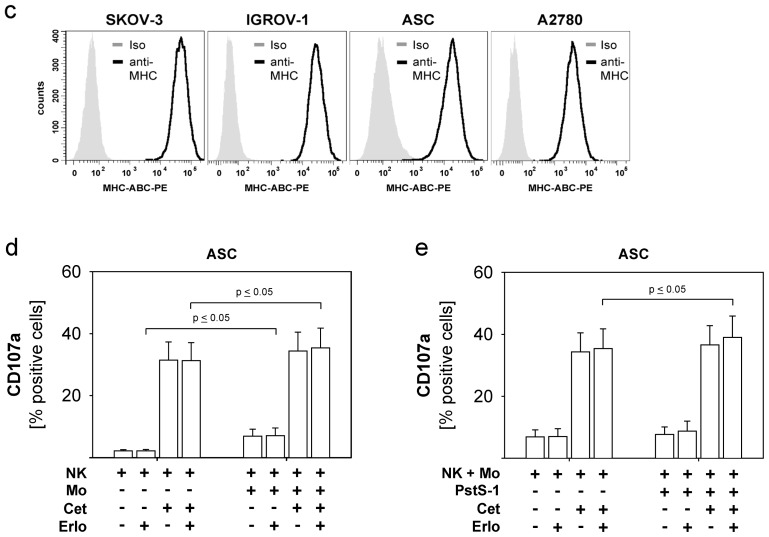
NK activity in the presence of monocytes, PstS-1, TKI and Cetuximab and correlation to MHC class I-expression. NK cells and monocytes of healthy donors were isolated by density gradient centrifugation and purified by MACS. NK cells were co-cultured with monocytes (5 × 10^5^ cells/well in 1:1 cell-ratio) and stimulated with PstS-1 (10 μg/mL). After 24 h Cetuximab (1 μg/mL) and TKI (100 nM) were added. NK cytotoxicity was assessed by CD107a degranulation assay after FITC-coupled staining with anti-CD107a and isotype control and gating on NK cells. Means and standard errors of three to six independent experiments are shown. Statistical analysis was performed by unpaired t-test, statistical significance (*p* < 0.05) is indicated. (**a**) Natural cytolytic activity of unstimulated NK cells (NK) against EGFR-positive and -negative ovarian cancer cells. (**b**) ADCC: CD107a-expression of NK cells co-incubated with ovarian cancer cells in the presence of Cetuximab (Cet) and TKI (Erlo); (**c**) MHC class I-expression of ovarian cells after staining with PE-coupled anti-MHC-class I and isotype control (Iso) analyzed by flow cytometry; (**d**) Effect of monocytes on NK-cytotoxicity in the presence or absence of Cetuximab and TKI; (**e**) Impact of PstS-1 on monocytes-stimulated NK cells (NK + Mo). CD107a-expression of monocytes-stimulated NK cells is moderately but significantly enhanced by PstS-1 in the presence of Cetuximab and TKI (39.3% ± 6.3%) compared to absence of PstS-1 (34.9% ± 6.8%).

As shown in Figure 4a degranulation of unstimulated NK cells against different EGFR-positive ovarian cancer cells (IGROV-1, ASC, SKOV-3) was only minimal, while the EGFR-negative cell line A2780 induced higher natural NK-cytotoxicity. This innate NK-resistance of EGFR-positive ovarian cancer cells could by overridden by the addition of Cetuximab (1 μg/mL) (NK + Cet) via an ADCC-mechanism, which resulted in a significant enhancement of NK-degranulation (Figure 4b: IGROV-1: *p* ≤ 0.01, ASC: *p* ≤ 0.01). This ADCC-effect was not impaired by addition of TKI (Erlo) in a concentration of 100 nM (Figure 4b: IGROV-1: *p* ≤ 0.01, ASC: *p* ≤ 0.01). This is of particular importance as related compounds as Dasatinib and Nilotinib negatively affected NK-functions [20]. However, Erlotinib, Gefitinib and Vandetanib could also not enhance susceptibility to NK-cytotoxicity. Further studies showed that in our collection of ovarian cancer cells the extent of NK-susceptibility was correlated to the expression level of MHC class I-molecules, as is illustrated in Figure 4c. While SKOV-3, IGROV-1 and ASC displayed high expression of MHC class I, A2780 expressed lower levels of MHC class I. In contrast, the level of the NKG2D-ligand MICA did not correlate to NK-cytotoxicity (data not shown). In addition, coincubation of TKI for the duration of CD107a-assay (6 h) altered neither MICA- nor MHC-expression (data not shown). As expected, NK-cytotoxicity against the EGFR-negative line A2780 could not be further enhanced by the addition of Cetuximab or TKI.

Based on our previous studies, which showed that PstS-1 and monocytes potentially enhance NK functions (manuscript submitted), we investigated whether cytolytic NK activity could be further augmented by additional monocytes and PstS-1 particularly in the presence of anti-EGFR-substances. To this end, NK cells were stimulated with monocytes +/− PstS-1 for 24 h. Cetuximab was added in a concentration of 1 μg/mL, while TKI was concentrated to 100 nM. NK-degranulation was determined by the expression of CD107a after coincubation with the cell lines IGROV-1, SKOV-3, A2780 and the primary culture ASC. Figure 4d shows the results for ASC, which is representative for the other lines being resistant to natural NK-cytotoxicity (NK) and anti-EGFR-substances. Natural NK-degranulation was significantly enhanced by the addition of monocytes (Mo) (*p* ≤ 0.05) and TKI did not impair this amplification. Similarly, the Cetuximab-mediated ADCC-effect was also augmented by monocytes (*p* ≤ 0.05) and was likewise not influenced by TKI. Figure 4e shows the ability of PstS-1 to enhance NK-cytotoxicity stimulated by monocytes against ASC-cells with or without Cetuximab and TKI. Our results show that monocytes-enhanced NK-cytotoxicity was moderately but significantly enhanced by PstS-1 in the presence of Cetuximab and TKI (*p* ≤ 0.05). Again, susceptibility of targets was not changed in the presence of TKI. Interestingly, PstS-1 did not augment cytolysis in the absence of cetuximab.

Our data indicate that natural NK-resistance of different ovarian cancer cells, which also show co-resistance to anti-EGFR targeted therapy, can be effectively overcome by the addition of the anti-EGFR-antibody Cetuximab. Addition of Cetuximab results in ADCC, which can be further effectively enhanced by accessory monocytes and the immunogenic molecule PstS-1. The additional presence of the anti-EGFR-TKI Erlotinib, Gefitinib and Vandetanib did not impair these amplifying mechanisms, which is in contrast to previous studies with related substances in other tumour entities [20]. On the other hand the anti-EGFR-TKI were unable to augment NK-cytotoxicity. Thus, at least in ovarian cancer, short-term application of anti-EGFR-TKI might not influence cross talk between tumour- and immune cells.

## 3. Experimental Section

### 3.1. Cell lines and Cell Culture

The human ovarian cancer cell lines A2780 and JA-I were obtained from *Westdeutsches Tumorzentrum*, University of Duisburg-Essen, Germany and cultured in RPMI-1640 and DMEM (high-glucose Dulbecco’s Modified Eagle Medium, Invitrogen) (3:1 *v*/*v*) supplemented with 10% FCS (Biochrom), 1% penicillin/streptomycin (Invitrogen) and 1% sodium pyruvate (Invitrogen). The cell lines IGROV-1, SKOV-3 and OVCAR-3 were provided by Dr. M. Mallmann, MD (*Life and Medical Sciences Institute*, University of Bonn, Germany) and cultured in RPMI-1640 supplemented with 10% FCS, 100 units/mL penicillin and 100 μg/mL streptomycin. To generate a primary ascites tumour cell culture (ASC), ascitic fluid was collected from a patient at first diagnosis for advanced low-differentiated (G3) serous ovarian cancer during debulking surgery (Department of Gynecology and Obstetrics, University of Duisburg-Essen). Written informed consent of the patient was obtained. Adherent ascitic tumour cells were cultured in RPMI-1640 and DMEM (3:1 *v*/*v*) supplemented with 10% FCS plus 1% penicillin/streptomycin and sodium pyruvate, continuously passaged and routinely inspected. Cultures were used when pure tumour cell growth was achieved and confirmed by microscopic examination. All tumour cells were incubated in plastic culture flasks (Greiner, Solingen, Germany) at 37 °C and 5% CO_2_ and continuously passaged by treatment with Accutase (PAA, Pasching, Austria) for 5 min at 37 °C.

### 3.2. MTT-Proliferation-Assay

To assess tumour cell viability under anti-EGFR-targeted therapy an MTT colorimetric assay was performed [29]. Tumour cell lines and the primary ascites culture were seeded into 96-well-plates (10,000 cells/well) and treated with the monoclonal anti-EGFR-antibody Cetuximab (Erbitux^®^, ImClone Systems, Bristol-Myers Squibb, New York, USA und Merck KGaA, Darmstadt, Germany) in concentrations of 1, 3, 10, 50 and 100 μg/mL. For IGROV-1 the antibody was further titrated to reach minimal inhibiting concentrations (0.5, 0.1 and 0.05 μg/mL). The anti-EGFR-TKIs Erlotinib, Gefitinib and Vandetanib (all from Selleck Chemicals, Houston, TX, USA) were reconstituted in DMSO (dimethyl sulfoxide, Carl-Roth-GmbH, Karlsruhe, Germany) before use. For the MTT-assay Erlotinib and Gefitinib were used in a concentration ranging from 1 μM to 1 nM, Vandetanib was concentrated between 1 μM and 10 nM. DMSO-controls were carried out. After 72 h at 37 °C and 5% CO_2_ cell viability was assessed using the MTT assay. MTT was added for three to five hours. Cells were lysed by DMSO and analyzed photometrically using six replicates per experimental condition. The percentage of cell viability was calculated by the following formula: [(Optical density of treated cells)/(Optical density of untreated cells) × 100].

### 3.3. Isolation of NK Cells and Monocytes from PBMC’s of Healthy Donors

All experiments were conducted with fresh purified blood cells from healthy donors and were immediately used after isolation. For isolation of mononuclear cells (MNCs) a density gradient centrifugation (Biocoll Separating Solution, Biochrom AG, Berlin, Germany) was performed at 25 °C, 300*g* for 30 min with whole blood diluted (1:1, *v*/*v*) with phosphate-buffered saline (PBS). The MNC fraction was collected, washed repeatedly with PBS and counted (CasyCounter; Innovatis-Roche, Bielefeld, Germany). NK cells and monocytes were isolated by the magnetic cell separator NK isolation kit II and CD14-beads (both from Miltenyi Biotec, Mönchengladbach, Germany) according to manufacturer’s protocol. Purity of cell subsets was routinely tested and ranged from 90% to 97%.

### 3.4. Stimulation of Purified NK Cells and Monocytes with PstS-1

Purified NK cells were cultured with or without monocytes (0.5 × 10^6^ cells/well, cell-cell-ratio 1:1), and stimulated with 10 μg/mL of PstS-1 (obtained from M. Singh, PhD, Lionex GmbH, Braunschweig, Germany) in a 24 well plate (Greiner Bio-One, Frickenhausen, Germany) for 24 h. CD107a degranulation assay was performed with or without Erlotinib and Cetuximab after addition of ovarian cancer target cells.

### 3.5. Flow Cytometric Analysis (FACS)

All cell lines including the primary ascitic cell culture were characterized regarding the expression of EGFR, MICA and MHC class I. For flow cytometry the following antibodies were used: anti-EGFR-PE (clone EGFR.1) and isotype mIgG2b-PE both from BD Bioscience, Heidelberg, Germany; anti-MICA (clone AMO1) from Immatics, Tübingen, Germany, unconjugated isotype mIgG1 (Dako, Hamburg, Germany) and secondary antibody goat-anti-mouse IgG1-PE (Dianova, Hamburg, Germany); anti-MHC class I-PE (clone W6/32) and isotype mIgG2a-PE both from Dako. For CD107a degranulation assay anti-CD107a-FITC (clone H4A3) and isotype mIgG1-FITC both from BD Bioscience were used. Cells were analyzed on a FACS Canto II using Diva software 6.0 (Becton Dickinson, Heidelberg, Germany).

### 3.6. CD107a Degranulation Assay and ADCC

The expression of CD107a was used to evaluate natural NK cell cytotoxicity and ADCC of unstimulated and stimulated NK cells [42]. Ovarian cancer cells (IGROV-1, A2780, SKOV-3, ASC) were coincubated with monocytes-/PstS-1-stimulated NK cells (cell-ratio 1:1) and unstimulated NK cells as controls on a flat-bottom 96-microtiter well plate (Greiner Bio-One). Cetuximab antibody was directly added at 1 μg/mL in ADCC experiments, the TKI’s Erlotinib, Gefitinib and Vandetanib were used in a concentration of 100 nM. NK cells were labeled with anti-CD107a-FITC or isotype mIgG1-FITC 1:20. After one hour incubation at 37 °C in 5% CO_2_ the golgi-stop monensin (BD Golgi-stop, BD Bioscience) was added at a dilution of 1:600. After further five hour incubation time cells were resuspended in 200 μL azide-PBS and immediately analyzed in the flow cytometer.

### 3.7. Flow Cytometric DNA-Staining and Cell Cycle Analysis

Procedure and analysis were performed according to the protocol of Riccardi *et al.* [30]. DNA extraction buffer was prepared by 192 mL Na_2_HPO_4_ (Merck KGaA GmbH) and 8 mL 0.1% Triton X-100 (Sigma) and adjusted to pH 7.8. DNA staining solution consisted of 200 μg Propidiumiodide (PI, Sigma) solved in 10 mL PBS. Two milligrams of DNAse free RNAase (Roche) was added per 10 mL staining solution. TKI- and Cetuximab-treated ovarian cancer cells and untreated controls were harvested, washed in PBS and fixed in 70% cold ethanol. Cells were washed twice with PBS, resuspended with DNA-extraction buffer and PBS (1:1 *v*/*v*) and incubated for five minutes at room temperature. After centrifugation at 400 g cells were resuspended with freshly prepared DNA-staining solution and incubated at least 30 min at room temperature in the dark. Cell cycle analysis was performed by flow cytometry using the 488-nm red line laser for excitation. Cell debris was gated off, doublet discrimination was done by gating on single cells in Area v Width dot plot of cells. Histograms of DNA-content showed the SubG1-, G0/G1-, S-, G2/M-phase of the cell cycle.

### 3.8. Statistical Analysis

Results are expressed as means and standard errors of several independent experiments. For statistical evaluation unpaired *t*-test was performed, statistical significance was assumed at level of *p* ≤ 0.05. The statistical calculations and illustrations were performed using SigmaPlot for Windows Version 11 (Systat Software GmbH, Erkrath, Germany).

## 4. Conclusions

In conclusion, this study shows that primary and long-term cultured ovarian cancer cells are largely resistant to anti-EGFR-targeted therapies, which correlates with the limited efficacy of clinical studies evaluating anti-EGFR-substances in ovarian cancer [8,10,13,17]. Even combined therapy consisting of monoclonal antibody and TKI failed to abrogate resistance in our model, which has not been studied in clinical trials of ovarian cancer yet. The primary resistance to natural NK cell cytotoxicity could be potentially overridden by the anti-EGFR-antibody Cetuximab, accessory monocytes and the immunogenic substance PstS-1. Additionally, the presence of anti-EGFR-TKI Erlotinib, Gefitinib and Vandetanib did not impair cytotoxic NK-functions. This might be of clinical relevance and is in contrast to observations with the related substances Dasatinib and Nilotinib in other tumour entities [20]. Future studies should explore the combined use of targeted therapy, adoptive NK cell transfer and immunostimulatory substances in patients with advanced ovarian cancer resistant to standard treatment.

## References

[1] Howlader N.A., Krapcho M., Neyman N., Aminou R., Altekruse S.F., Kosary C.L., Ruhl J., Tatalovich Z., Cho H., Mariotto A. Seer cancer statistics review, 1975–2009 (vintage 2009 populations).

[2] Bartlett J.M., Langdon S.P., Simpson B.J., Stewart M., Katsaros D., Sismondi P., Love S., Scott W.N., Williams A.R., Lessells A.M. (1996). The prognostic value of epidermal growth factor receptor mRNA expression in primary ovarian cancer. Br. J. Cancer.

[3] Fischer-Colbrie J., Witt A., Heinzl H., Speiser P., Czerwenka K., Sevelda P., Zeillinger R. (1997). EGFR and steroid receptors in ovarian carcinoma: Comparison with prognostic parameters and outcome of patients. Anticancer Res.

[4] Mendelsohn J., Baselga J. (2003). Status of epidermal growth factor receptor antagonists in the biology and treatment of cancer. J. Clin. Oncol.

[5] Bijman M.N., van Berkel M.P., Kok M., Janmaat M.L., Boven E. (2009). Inhibition of functional HER family members increases the sensitivity to docetaxel in human ovarian cancer cell lines. AntiCancer Drugs.

[6] Konner J., Schilder R.J., DeRosa F.A., Gerst S.R., Tew W.P., Sabbatini P.J., Hensley M.L., Spriggs D.R., Aghajanian C.A. (2008). A phase II study of cetuximab/paclitaxel/carboplatin for the initial treatment of advanced-stage ovarian, primary peritoneal, or fallopian tube cancer. Gynecol. Oncol.

[7] Secord A.A., Blessing J.A., Armstrong D.K., Rodgers W.H., Miner Z., Barnes M.N., Lewandowski G., Mannel R.S., Gynecologic Oncology Group (2008). Phase II trial of cetuximab and carboplatin in relapsed platinum-sensitive ovarian cancer and evaluation of epidermal growth factor receptor expression: A gynecologic oncology group study. Gynecol. Oncol.

[8] Schilder R.J., Pathak H.B., Lokshin A.E., Holloway R.W., Alvarez R.D., Aghajanian C., Min H., Devarajan K., Ross E., Drescher C.W. (2009). Phase II trial of single agent cetuximab in patients with persistent or recurrent epithelial ovarian or primary peritoneal carcinoma with the potential for dose escalation to rash. Gynecol. Oncol.

[9] Gordon A.N., Finkler N., Edwards R.P., Garcia A.A., Crozier M., Irwin D.H., Barrett E. (2005). Efficacy and safety of erlotinib HCl, an epidermal growth factor receptor (HER-1/EGFR) tyrosine kinase inhibitor, in patients with advanced ovarian carcinoma: Results from a phase II multicenter study. Int. J. Gynecol. Cancer.

[10] Blank S.V., Christos P., Curtin J.P., Goldman N., Runowicz C.D., Sparano J.A., Liebes L., Chen H.X., Muggia F.M. (2010). Erlotinib added to carboplatin and paclitaxel as first-line treatment of ovarian cancer: A phase II study based on surgical reassessment. Gynecol. Oncol.

[11] Vasey P.A., Gore M., Wilson R., Rustin G., Gabra H., Guastalla J.P., Lauraine E.P., Paul J., Carty K., Kaye S., Scottish Gynecological Cancer Trials Group (2008). A phase Ib trial of docetaxel, carboplatin and erlotinib in ovarian, fallopian tube and primary peritoneal cancers. Br. J. Cancer.

[12] Chambers S.K., Clouser M.C., Baker A.F., Roe D.J., Cui H., Brewer M.A., Hatch K.D., Gordon M.S., Janicek M.F., Isaacs J.D. (2010). Overexpression of tumour vascular endothelial growth factor A may portend an increased likelihood of progression in a phase II trial of bevacizumab and erlotinib in resistant ovarian cancer. Clin. Cancer Res.

[13] Schilder R.J., Sill M.W., Chen X., Darcy K.M., Decesare S.L., Lewandowski G., Lee R.B., Arciero C.A., Wu H., Godwin A.K. (2005). Phase II study of gefitinib in patients with relapsed or persistent ovarian or primary peritoneal carcinoma and evaluation of epidermal growth factor receptor mutations and immunohistochemical expression: A Gynecologic Oncology Group study. Clin. Cancer Res.

[14] Posadas E.M., Liel M.S., Kwitkowski V., Minasian L., Godwin A.K., Hussain M.M., Espina V., Wood B.J., Steinberg S.M., Kohn E.C. (2007). A phase II and pharmacodynamic study of gefitinib in patients with refractory or recurrent epithelial ovarian cancer. Cancer.

[15] Ciardiello F., Caputo R., Bianco R., Damiano V., Pomatico G., de Placido S., Bianco A.R., Tortora G. (2000). Antitumour effect and potentiation of cytotoxic drugs activity in human cancer cells by ZD-1839 (Iressa), an epidermal growth factor receptor-selective tyrosine kinase inhibitor. Clin. Cancer Res.

[16] Pautier P., Joly F., Kerbrat P., Bougnoux P., Fumoleau P., Petit T., Rixe O., Ringeisen F., Carrasco A.T., Lhomme C. (2010). Phase II study of gefitinib in combination with paclitaxel (p) and carboplatin (c) as second-line therapy for ovarian, tubal or peritoneal adenocarcinoma (1839IL/0074). Gynecol. Oncol.

[17] Annunziata C.M., Walker A.J., Minasian L., Yu M., Kotz H., Wood B.J., Calvo K., Choyke P., Kimm D., Steinberg S.M. (2010). Vandetanib, designed to inhibit VEGFR2 and EGFR signaling, had no clinical activity as monotherapy for recurrent ovarian cancer and no detectable modulation of VEGFR2. Clin. Cancer Res.

[18] Zhang W., Gordon M., Schultheis A.M., Yang D.Y., Nagashima F., Azuma M., Chang H.M., Borucka E., Lurje G., Sherrod A.E. (2007). FCGR2a and FCGR3a polymorphisms associated with clinical outcome of epidermal growth factor receptor expressing metastatic colorectal cancer patients treated with single-agent cetuximab. J. Clin. Oncol.

[19] Screpanti V., Wallin R.P., Grandien A., Ljunggren H.G. (2005). Impact of FasL-induced apoptosis in the elimination of tumour cells by NK cells. Mol. Immunol.

[20] Salih J., Hilpert J., Placke T., Grunebach F., Steinle A., Salih H.R., Krusch M. (2010). The Bcr/abl-inhibitors imatinib, nilotinib and dasatinib differentially affect NK cell reactivity. Int. J. Cancer.

[21] Lamm D.L., Blumenstein B.A., Crawford E.D., Montie J.E., Scardino P., Grossman H.B., Stanisic T.H., Smith J.A., Sullivan J., Sarosdy M.F. (1991). A randomized trial of intravesical doxorubicin and immunotherapy with bacille calmette-guerin for transitional-cell carcinoma of the bladder. N. Engl. J. Med..

[22] Brandau S., Riemensberger J., Jacobsen M., Kemp D., Zhao W., Zhao X., Jocham D., Ratliff T.L., Bohle A. (2001). NK cells are essential for effective BCG immunotherapy. Int. J. Cancer.

[23] Suttmann H., Jacobsen M., Reiss K., Jocham D., Bohle A., Brandau S. (2004). Mechanisms of bacillus calmette-guerin mediated natural killer cell activation. J. Urol.

[24] Singh M., Andersen A.B., McCarthy J.E., Rohde M., Schutte H., Sanders E., Timmis K.N. (1992). The mycobacterium tuberculosis 38-kDa antigen: Overproduction in escherichia coli, purification and characterization. Gene.

[25] Rodriguez A., Troye-Blomberg M., Lindroth K., Ivanyi J., Singh M., Fernandez C. (2003). B- and T*-*cell responses to the mycobacterium surface antigen PstS-1 in the respiratory tract and adjacent tissues. Role of adjuvants and routes of immunization. Vaccine.

[26] Sanger C., Busche A., Bentien G., Spallek R., Jonas F., Bohle A., Singh M., Brandau S. (2004). Immunodominant PstS-1 antigen of mycobacterium tuberculosis is a potent biological response modifier for the treatment of bladder cancer. BMC Cancer.

[27] Jung S.B., Yang C.S., Lee J.S., Shin A.R., Jung S.S., Son J.W., Harding C.V., Kim H.J., Park J.K., Paik T.H. (2006). The mycobacterial 38-kilodalton glycolipoprotein antigen activates the mitogen-activated protein kinase pathway and release of proinflammatory cytokines through Toll-like receptors 2 and 4 in human monocytes. Infect. Immun.

[28] Sanchez A., Espinosa P., Esparza M.A., Colon M., Bernal G., Mancilla R. (2009). Mycobacterium tuberculosis 38-kda lipoprotein is apoptogenic for human monocyte-derived macrophages. Scand. J. Immunol.

[29] Mosmann T. (1983). Rapid colorimetric assay for cellular growth and survival: Application to proliferation and cytotoxicity assays. J. Immunol. Methods.

[30] Riccardi C., Nicoletti I. (2006). Analysis of apoptosis by propidium iodide staining and flow cytometry. Nature Protoc.

[31] Prewett M., Rockwell P., Rose C., Goldstein N. (1996). Anti-tumour and cell cycle responses in KB cells treated with a chimeric anti-EGFR monoclonal antibody in combination with cisplatin. Int. J. Oncol.

[32] Karapetis C.S., Khambata-Ford S., Jonker D.J., O’Callaghan C.J., Tu D., Tebbutt N.C., Simes R.J., Chalchal H., Shapiro J.D., Robitaille S. (2008). K-ras mutations and benefit from cetuximab in advanced colorectal cancer. N. Engl. J. Med.

[33] Wheeler D.L., Huang S., Kruser T.J., Nechrebecki M.M., Armstrong E.A., Benavente S., Gondi V., Hsu K.T., Harari P.M. (2008). Mechanisms of acquired resistance to cetuximab: Role of HER (ErbB) family members. Oncogene.

[34] Grunt T.W., Wagner R., Grusch M., Berger W., Singer C.F., Marian B., Zielinski C.C., Lupu R. (2009). Interaction between fatty acid synthase- and ErbB-systems in ovarian cancer cells. Biochem. Biophys. Res. Commun.

[35] Lynch T.J., Bell D.W., Sordella R., Gurubhagavatula S., Okimoto R.A., Brannigan B.W., Harris P.L., Haserlat S.M., Supko J.G., Haluska F.G. (2004). Activating mutations in the epidermal growth factor receptor underlying responsiveness of non-small-cell lung cancer to gefitinib. N. Engl. J. Med.

[36] Bull Phelps S.L., Schorge J.O., Peyton M.J., Shigematsu H., Xiang L.L., Miller D.S., Lea J.S. (2008). Implications of EGFR inhibition in ovarian cancer cell proliferation. Gynecol. Oncol.

[37] Tanaka Y., Terai Y., Tanabe A., Sasaki H., Sekijima T., Fujiwara S., Yamashita Y., Kanemura M., Ueda M., Sugita M. (2011). Prognostic effect of epidermal growth factor receptor gene mutations and the aberrant phosphorylation of Akt and ERK in ovarian cancer. Cancer Biol. Ther.

[38] Huether A., Hopfner M., Baradari V., Schuppan D., Scherubl H. (2005). EGFR blockade by cetuximab alone or as combination therapy for growth control of hepatocellular cancer. Biochem. Pharmacol.

[39] Huang S., Armstrong E.A., Benavente S., Chinnaiyan P., Harari P.M. (2004). Dual-agent molecular targeting of the epidermal growth factor receptor (EGFR): Combining anti-EGFR antibody with tyrosine kinase inhibitor. Cancer Res.

[40] Janjigian Y.Y., Azzoli C.G., Krug L.M., Pereira L.K., Rizvi N.A., Pietanza M.C., Kris M.G., Ginsberg M.S., Pao W., Miller V.A. (2011). Phase I/II trial of cetuximab and erlotinib in patients with lung adenocarcinoma and acquired resistance to erlotinib. Clin. Cancer Res.

[41] Weickhardt A.J., Price T.J., Chong G., Gebski V., Pavlakis N., Johns T.G., Azad A., Skrinos E., Fluck K., Dobrovic A. (2012). Dual targeting of the epidermal growth factor receptor using the combination of cetuximab and erlotinib: Preclinical evaluation and results of the phase II DUX study in chemotherapy-refractory, advanced colorectal cancer. J. Clin. Oncol.

[42] Alter G., Malenfant J.M., Altfeld M. (2004). CD107a as a functional marker for the identification of natural killer cell activity. J. Immunol. Methods.

